# Hepatic stellate cell hypertrophy is associated with metabolic liver fibrosis

**DOI:** 10.1038/s41598-020-60615-0

**Published:** 2020-03-02

**Authors:** Céline Hoffmann, Nour El Houda Djerir, Anne Danckaert, Julien Fernandes, Pascal Roux, Christine Charrueau, Anne-Marie Lachagès, Frédéric Charlotte, Isabelle Brocheriou, Karine Clément, Judith Aron-Wisnewsky, Fabienne Foufelle, Vlad Ratziu, Bernard Hainque, Dominique Bonnefont-Rousselot, Pascal Bigey, Virginie Escriou

**Affiliations:** 1 Université Paris Descartes, UTCBS, CNRS, INSERM, F-75006 Paris, France; 2 Institut Pasteur, C2RT, UTechS Photonic BioImaging, Paris, France; 3 Service d’Anatomie et Cytologie Pathologiques, Hôpitaux universitaires Pitié-Salpêtrière-Charles Foix (AP-HP), F-75013 Paris, France; 4 Sorbonne Université, Inserm, UMRS NutriOmique, Service de Nutrition, Hôpitaux universitaires Pitié-Salpêtrière-Charles Foix, F-75013 Paris, France; 5 INSERM, Sorbonne Université, Université de Paris; Centre de Recherche des Cordeliers, F-75006 Paris, France; 6 Sorbonne Université, Institute for Cardiometabolism and Nutrition, Hôpital Pitié Salpêtrière, Assistance Publique Hôpitaux de Paris, Paris, France; 7 Service de Biochimie métabolique, Hôpitaux universitaires Pitié-Salpêtrière-Charles Foix (AP-HP), F-75013 Paris, France; 8 Chimie ParisTech, PSL Research University, UTCBS, F-75005 Paris, France

**Keywords:** Liver fibrosis, Non-alcoholic steatohepatitis, Hepatic stellate cells

## Abstract

Hepatic fibrosis is a major consequence of chronic liver disease such as non-alcoholic steatohepatitis which is undergoing a dramatic evolution given the obesity progression worldwide, and has no treatment to date. Hepatic stellate cells (HSCs) play a key role in the fibrosis process, because in chronic liver damage, they transdifferentiate from a “quiescent” to an “activated” phenotype responsible for most the collagen deposition in liver tissue. Here, using a diet-induced liver fibrosis murine model (choline-deficient amino acid-defined, high fat diet), we characterized a specific population of HSCs organized as clusters presenting simultaneously hypertrophy of retinoid droplets, quiescent and activated HSC markers. We showed that hypertrophied HSCs co-localized with fibrosis areas in space and time. Importantly, we reported the existence of this phenotype and its association with collagen deposition in three other mouse fibrosis models, including CCl_4_-induced fibrosis model. Moreover, we have also shown its relevance in human liver fibrosis associated with different etiologies (obesity, non-alcoholic steatohepatitis, viral hepatitis C and alcoholism). In particular, we have demonstrated a significant positive correlation between the stage of liver fibrosis and HSC hypertrophy in a cohort of obese patients with hepatic fibrosis. These results lead us to conclude that hypertrophied HSCs are closely associated with hepatic fibrosis in a metabolic disease context and may represent a new marker of metabolic liver disease progression.

## Introduction

Hepatic fibrosis is a wound-healing response to long-term liver injury, including chronic viral infection, excessive alcohol consumption, non-alcoholic steatohepatitis (NASH) or autoimmune liver disease^1^. Hepatic fibrosis corresponds to the excessive accumulation of extracellular matrix (ECM) proteins, collagen fibers in particular, in the liver parenchyma that distorts liver architecture by forming a fibrous scar. Advanced hepatic fibrosis leads to cirrhosis, which results in hepatocellular dysfunction and increased intrahepatic resistance to blood flow, and often requires liver transplantation. A better understanding of fibrogenesis and resolution of fibrosis has revealed a number of potential antifibrotic targets^2^. Although many drug candidates have been tested in preclinical models and many of them are in human clinical trials, none are currently approved by the FDA (Food and drug administration).

Hepatic stellate cells (HSCs), portal fibroblasts, and myofibroblasts of bone marrow origin have been identified as the major extracellular matrix protein producing cells in the injured liver^3–5^. Among all, HSCs are recognized as the major contributing cells in the fibrosis process: in the presence of chronic inflammation, they transdifferentiate from quiescent state into activated state showing a proliferative myofibroblast phenotype, which accounts for the major source of collagen expression and deposition in the liver tissue^6^. Given the primary role of the activated HSCs in fibrogenesis, they remain central to the development of antifibrotic therapy.

HSCs are localized in the space of Disse, interposed between liver sinusoidal endothelial cells (LSECs) and hepatocytes; they represent ~10% of all resident liver cells. In the normal liver, HSCs maintain a non-proliferative, quiescent phenotype with the main known function of storing vitamin A as retinyl esters into cytoplasmic lipid droplets^7^. The sequence of HSC activation describes a first step of ‘initiation’ that refers to early events like development of a contractile and fibrogenic phenotype and modulation of growth factor signaling, and a second ‘perpetuation’ step characterized by the amplification of the activated phenotype^8^. One major characteristic of activated HSCs is their loss of retinoid store^7^, but today, the contribution of stored retinoids to HSC activation and hepatic fibrosis still remains poorly understood.

Improving our understanding of the pathogenesis of liver fibrosis is based primarily on robust and reproducible experimental animal models. Repetition of a toxic attack on the liver is a classic way of inducing hepatic fibrosis in laboratory animals. One of the most widely used liver toxins for experimental induction of a liver injury is carbon tetrachloride (CCl_4_)^9^. Although this kind of hepatotoxic model reproduces some important features of human liver fibrosis, the etiology of the fibrosis is different from that resulting from a chronic metabolic liver disease such as NASH. To recapitulate metabolic liver disease, other models such as nutritional models may be used. Methionine and choline deficient (MCD) diet is the major nutritional model for NASH study and induces macrovesicular steatosis, hepatocellular death, inflammation, oxidative stress, and liver fibrosis^10^. However, a disadvantage of using this diet is that mice exhibit dramatic weight loss associated with an increased risk of death limiting the duration of the experiments and the level of hepatic fibrosis obtained. Interestingly, it has been shown that supplementation of 0.1% methionine and high fat in MCD diet, called choline-deficient amino acid-defined and high fat diet (CDAHFD), is able to overcome some of these drawbacks and has several advantages, including that of inducing marked fibrosis, appearing earlier than in other nutritional models, with high inter-individual reproducibility and no mortality^11^.

While using CDAHFD mouse model in a parallel study to investigate the role of HSCs in the onset and development of hepatic fibrosis in a steatohepatitis context, we found out the existence of clusters of HSCs, characterized by a particular phenotype not previously described in a context of metabolic liver fibrosis.

This paper describes the characterization of this specific population of HSCs and focuses on demonstrating its association with hepatic fibrosis, on several animal models of metabolic liver fibrosis, as well as in a preliminary study on human samples exhibiting liver fibrosis.

## Results

### CDAHFD induces steatosis, ballooning, inflammation and progressive liver fibrosis

**Figure 1:**
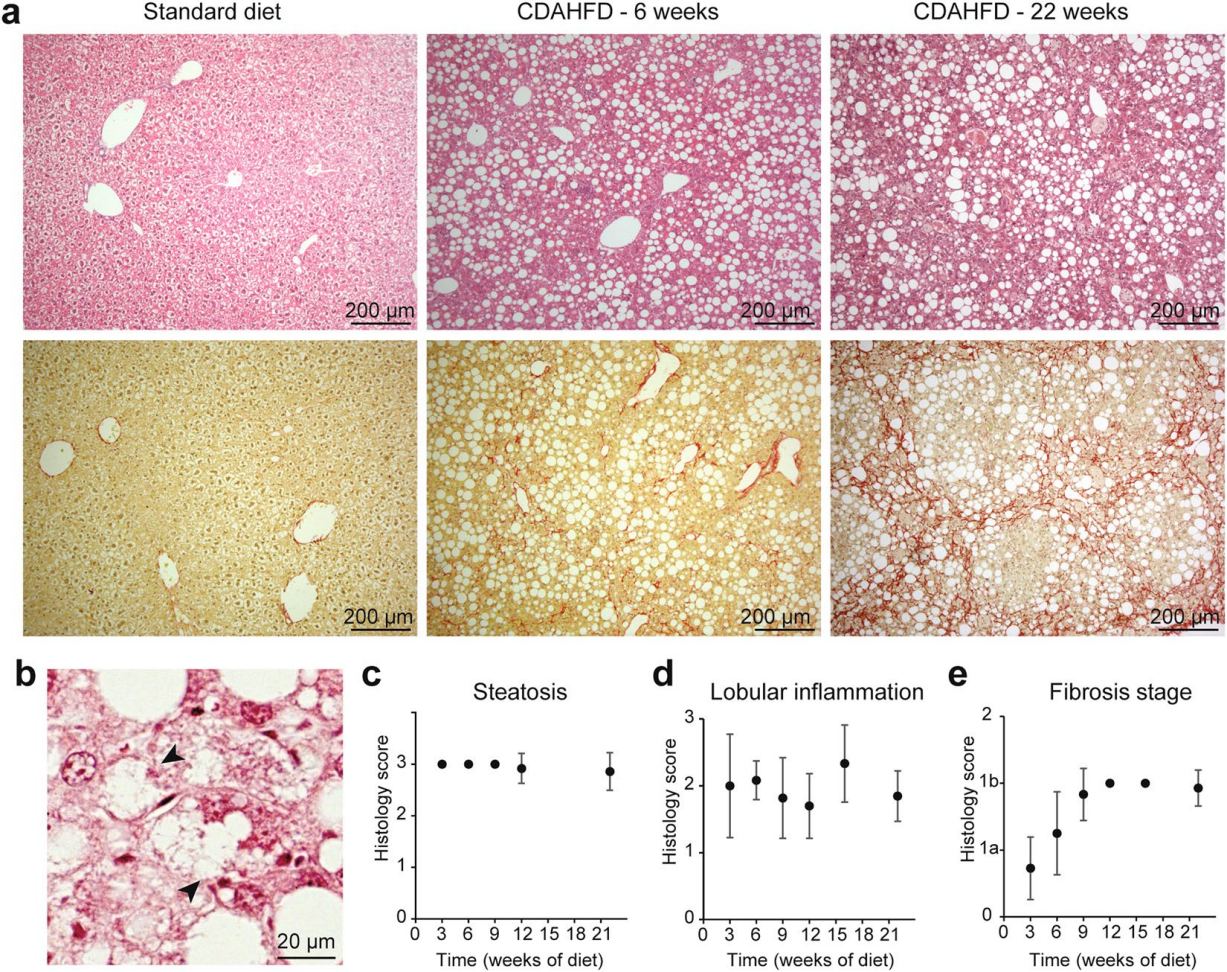
Histological characterization of CDAHFD mice model. (**a**) Histological colorations (top: Hematoxylin/Eosin, low: Sirius Red) of Standard Diet- or CDAHFD-fed mice for 6 or 22 weeks. **(b**) Example of hepatocyte ballooning in a CDAHFD-fed mice (arrowheads). **(c–e**) Histological scores: steatosis (**c**), inflammation (**d)** and fibrosis (**e**) as a function of time. The points corresponding to 3, 6 and 9 weeks in (**c**) and 12 and 15 weeks in **(e**) are not associated with an error bar because for these points, the values of the scores are identical and the standard deviation is therefore 0. (n = 10–14 for each time point, means ± standard deviation).

We conducted histopathological assessment of diet-induced liver lesions. Figure 1a shows representative micrographs of Hematoxylin & Eosin (H&E) coloration and Sirius Red stain of mice liver sections after feeding with standard diet (SD) or CDAHFD for 6 and 22 weeks. Steatosis reached the maximum score of 3 from the 3^rd^ week of CDAHFD (Fig. 1c). A grade 2 for lobular inflammation, characterized by inflammatory foci, was observed from the 3^rd^ week and maintained throughout the diet administration (Fig. 1d). Hepatocyte ballooning was also noticed in the livers of CDAHFD-fed mice (Fig. 1b), but not in the livers of SD-fed mice. Moreover, a slight perisinusoidal fibrosis was detected from the 6^th^ week of CDAHFD administration, which started from the centrilobular zone and evolved progressively to a marked stage from 9 weeks and then stabilized throughout the 22 weeks of experiment (Fig. 1e). These liver damages were particularly reproducible since they were found in all CDAHFD-fed mice. The metabolic abnormalities seen in mice fed with CDAHFD for 22 weeks were mainly significant increases in liver weight and in the markers for liver injury, ALT (Alanine transaminase) and AST (Aspartate transaminase) and consistent with the characterization of the model, as previously reported^11^.

### Hypertrophied cells located near collagen fibers occurred in fibrotic livers

**Figure 2:**
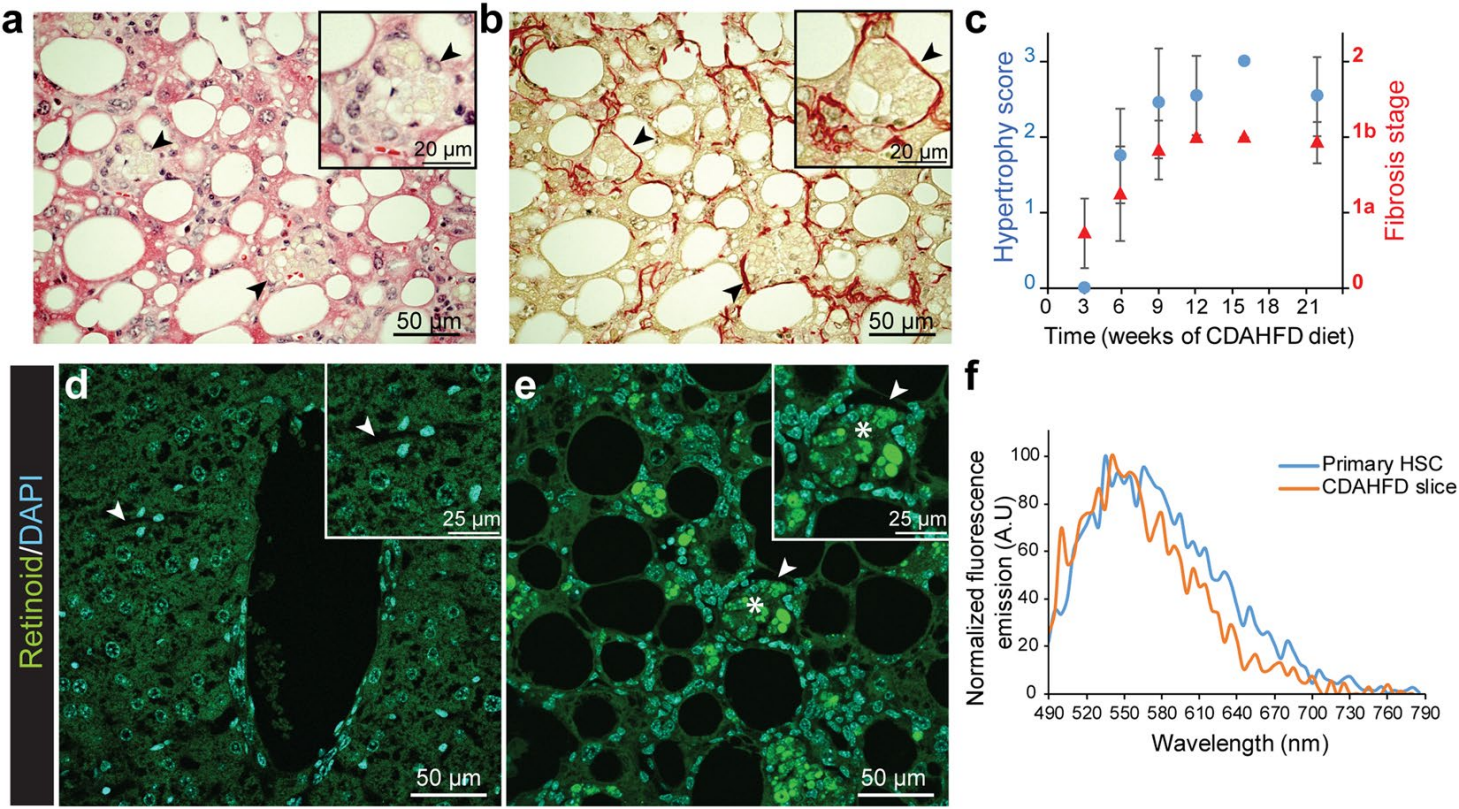
Hypertrophied cells are collocated with collagen fibers and full of fluorescent droplets. (**a,b**) Collocation (arrowheads) of hypertrophied cells (**a**, HE staining) and collagen fibers (**b**, Sirius Red staining) at 9 weeks of CDAHFD. (**c**) Cell hypertrophy (blue dots) and fibrosis (red triangles) scores as a function of CDAHFD time. (n = 10–14 for each time point, means ± standard deviation). (**d,e**) Combined images of DAPI (cyan, λ_exc_ = 405 nm) and retinoid (green, λ_exc_ = 488 nm) fluorescent signals on 9 weeks SD- (**d**) and CDAHFD-fed mice (**e**) liver slices. (Arrowheads: sinusoidal space, stars: retinoid fluorescent droplets). **(f**) Normalized fluorescence emission spectra of retinoid droplets in primary murine HSCs (blue) and 12 weeks CDAHFD-fed mouse liver slice (orange), measured by confocal microscopy (λ_exc_ = 480 nm, λ_em_ = 490–790 nm).

Further examination of H&E stained slices revealed cells with a swollen cytoplasm and enlarged by yellow droplets (Fig. 2a arrowheads). These cells, which can be described as hypertrophied, were mainly located in the perisinusoidal space. A score was set to quantify this occurrence by observation at 200x magnification, as follows: 0 = no visible hypertrophy, 1 = small and few hypertrophied cells are visible, 2 = bigger and less than 10 hypertrophied cells per microscopic field, 3 = more than 10 hypertrophied cells visible per microscopic field. This histological feature was detectable from the 6^th^ week of CDAHFD with a high reproducibility (100% of CDAHFD-fed mice exhibited hypertrophied cells), and increased progressively, following the same kinetics as that of fibrosis (Fig. 2c). In addition, analysis of Sirius red-stained sections showed that hypertrophied cells were close to collagen deposition (Fig. 2b, arrowheads). Because of the perisinusoidal localization of the hypertrophied cells as well as their proximity to the collagen fibers, it is reasonable to assume that these cells are HSCs. One of the typical features of HSCs is to contain droplets of retinoids with a well-characterized natural fluorescence^12^. Then, to further characterize the hypertrophied cells, we performed fluorescence microscopy analysis on liver slices of SD or CDAHFD-fed mice. On CDAHFD slices, cells located in the perisinusoidal space with strong fluorescent droplet accumulations were observed (Fig. 2e), whereas no fluorescent signal was detected on liver slices from SD-fed mice (Fig. 2d). These fluorescent areas detected on CDAHFD sections corresponded to the hypertrophied cells observed by widefield microscopy (Fig. 2a, arrowhead). Microscopic fluorescent spectral analysis allowed us to analyze these fluorescent droplets on CDAHFD liver slices. A characteristic fluorescence spectrum of retinoids was obtained after excitation (λ_exc_ = 480 nm) of droplets on CDAHFD-fed mouse liver slice and was superimposed with the one obtained on droplets of primary HSCs purified from SD-fed mouse, that confirmed that they were indeed retinoid droplets (Figs. 2f and S1).

These results show that the hypertrophy that we identified on liver cells of CDAHFD-fed mice is due to an increased accumulation of retinoid droplets in their cytoplasm.

### Further characterization of retinoid accumulation

**Figure 3:**
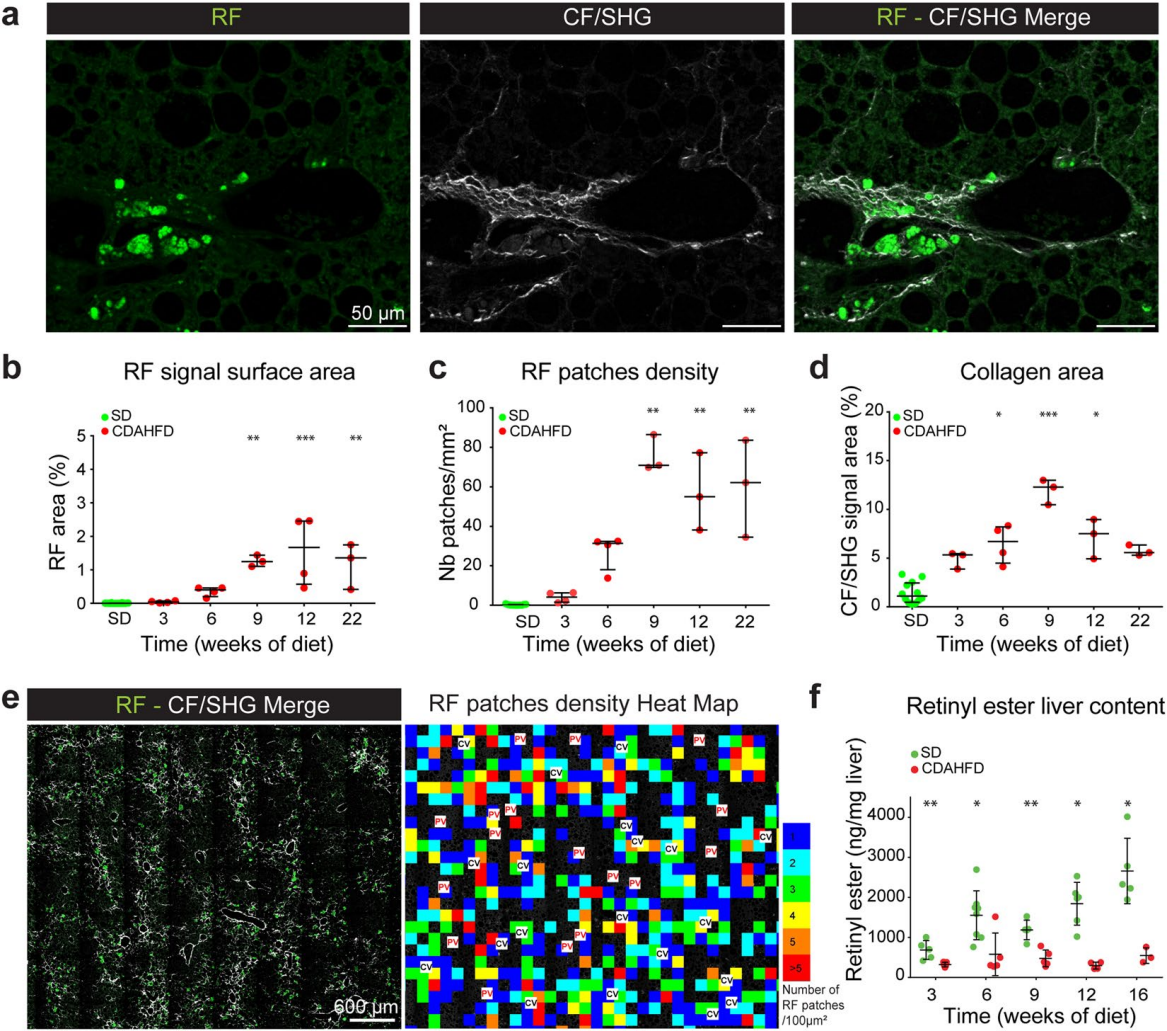
Spatio-temporal analysis of retinoid fluorescence (RF) and collagen fibers (CF/SHG). (**a**) Example of collocation of retinoid fluorescence (RF) imaged by fluorescence (left) and collagen fibers imaged by second harmonic generation (CF/SHG) (middle) microscopy. Right: merge of RF and CF/SHG signals. (**b**) Percentage of RF signal surface area on total SD- or CDAHFD-liver slices as a function of time. (**c**) RF patches density (number of RF patches per mm²) of total SD- or CDAHFD-liver slices as a function of time. (**d**) Percentage of CF/SHG signal surface area on total SD- or CDAHFD-liver slices as a function of time. (**b–d**) Statistics: n = 3–12 for each group, median with interquartile range, Kruskal Wallis test. (**e**) Merge of RF and CF/SHG signals of 9 weeks CDAHFD-fed mouse liver slice (left). Heat map of RF patches density of the same liver slice (right). Color scale  = number of RF patches for a 100 × 100 µm square. PV = Portal Vein, CV = Centrilobular Vein. (**f**) Total liver content in retinyl esters measured by HPLC as a function of time of SD or CDAHFD. (n = 4–7 for each condition, means ± standard deviation, Mann-Whitney test). *p < 0.05; **p < 0.01; ***p < 0.001.

We next performed multi-photon microscopy analysis, to detect both the fluorescence of retinoids, termed RF (retinoid fluorescence), and the specific signal of collagen fibers (CF) by second harmonic generation (SHG) microscopy, termed CF/SHG, on the same slice without any treatment. The RF signal corresponding to the accumulation of fluorescent retinoid droplets was closely located to the CF/SHG signal, indicative of collagen fiber deposition (Fig. 3a). Both RF and CF/SHG signals kinetics evolved concomitantly throughout the duration of diet administration (Supplementary Fig. S2), that is consistent with the observation we made previously with classical staining methods (Fig. 2a,b).

Using a pipeline of multiphoton microscopy analysis that we developed, we then carried out a spatio-temporal study of RF and CF/SHG signals. Image analysis was performed with an “in-house” developed macro under FiJi environment. For that, 3 to 22 weeks SD- or CDAHFD-fed mice liver slices were entirely scanned by multiphotonic microscopy both for RF and CF/SHG signals. Depending on its size, the RF signal may correspond to a droplet, several droplets indistinguishable within the same cell or a cluster of cells loaded with droplets. RF signal is processed in order to individualize each RF areas as “patches”, making it possible to count them and to determine the surface of each of them. Regions of interest (ROI) of CF/SHG signal were identified and quantified. Three parameters were obtained from these analyses and plotted as a function of diet time: the percentage of slice area covered by RF signal (Fig. 3b), by CF/SHG signal (Fig. 3d) and the number of RF patches by slice surface unit, or RF density (Fig. 3c). Under CDAHFD, RF signal was detected from the 6^th^ week, while under SD, it was undetectable regardless of the time of diet. RF area as well as RF density increased gradually from week 3 to week 9 of CDAHFD and stabilized until the end of the experiment (22 weeks) with a median of 1.17% of the total surface area (Fig. 3b) and 62 RF patches per mm² (n = 3) (Fig. 3c). Interestingly, analysis of the RF patches ‘size distribution showed that it evolved progressively during CDAHFD administration (Supplementary Fig. S2) showing a gradual increase of frequency of the larger RF patches. Concerning the CF/SHG signal (Fig. 3d), under SD, a basal signal was measured and maintained between 0.2 and 2.5% of the total area of the slice. This is consistent with the presence of collagen around veins and arteries visible in Supplementary Fig. S2 for example. Under CDAHFD, CF/SHG signal increased from a median of 5.3% at 3 weeks of diet and evolved progressively until 9 weeks of diet to attain a median of 12.3% of the total slice area. The algorithm we designed allowed us to analyze the spatial repartition of RF and CF/SHG signals. As shown in Fig. 3e, marked perisinusoidal fibrosis was observed in the liver centrilobular areas without invading the periportal areas. RF accumulation was highly concentrated in the centrilobular area as shown by the HeatMap on Fig. 3e. Hence, multiphoton microscopy allowed us both to highlight the onset of retinoid droplet-containing hypertrophied cells associated with collagen deposition and to quantify their simultaneous progression as a function of diet time.

This result is however contradictory with the accepted concept that HSCs lose their retinoid droplets when they differentiate into myofibroblasts and produce collagen. We then quantified, using retinoid extraction and HPLC (High-Performance Liquid Chromatography) analysis, the total amount of retinyl esters (retinoid storage form in HSCs) and retinol (free form of retinoids in liver) present in the liver of SD- or CDAHFD-fed mice at different times. Under SD, the level of retinyl esters tended to increase with time, whereas it was significantly lower under CDAHFD, as early as 3 weeks and throughout the experiment (Fig. 3f). The same result was observed for retinol (Supplementary Fig. S3). Overall, mice lost their retinoid stock that is consistent with the dogma of retinol loss by HSCs at the time of their activation^7^. Considering the total liver assay result, it can be proposed that in SD-fed mice, retinyl esters are stored in small amounts in HSCs, undetectable by fluorescence in thin liver sections, because signal is below the fluorescence detection limit. Interestingly, detection of RF become possible, on thicker (50 µm) sections of SD-fed mice liver (data not shown). In CDAHFD-fed mice, during establishment of fibrosis, HSCs activate and lose their retinoid storage leading to the decrease of the overall liver retinoid quantity. However, some cells would retain the remaining retinoids in particular areas where retinol will be “over-concentrated” in hypertrophied cells.

### Expression of hepatic stellate cells specific markers

**Figure 4:**
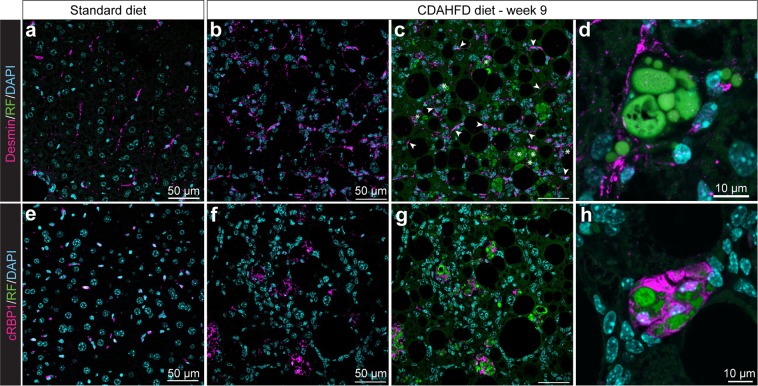
Expression of specific HSC markers in hypertrophy areas. (**a–d**) Desmin labelling of 9 weeks SD- (**a**) and CDAHFD-fed mice (**b**–**d**) liver slices. Overlap of DAPI, RF and desmin signals (**a,c,d**) or DAPI and desmin signal (**b**). Arrowheads: desmin-positive cells. Stars: desmin- and retinoid-positive cells. **(e–h**) cRBP1 labelling of 9 weeks SD- (**e**) and CDAHFD-fed mice (**f–h**) liver slices. Overlap of DAPI, RF and cRBP1 signals (**e,g,h**) or DAPI and cRBP1 (**f**).

To further characterize these areas of hypertrophied cells, we investigated the expression of HSC markers: desmin, an intermediate filament expressed in HSCs regardless of their activation status^13^, and cRBP1 (cellular retinol binding protein-1), a protein involved in the transport of retinol and strongly expressed in HSCs^14^ (Fig. 4). On SD slices, an elongated shape labelling of desmin was detected on cells along the perisinusoidal space (Fig. 4a). On CDAHFD slices, desmin labelling was detected at a greater frequency, showing together an elongated shape and a more rounded shape (Fig. 4b). The overlap of desmin and RF signals showed two populations of desmin-positive cells, one population that presented also fluorescent droplets of retinoids (stars, Fig. 4c), and another population that was positive for desmin with no RF (arrowheads, Fig. 4c). Both desmin-positive populations were observable from the 3^rd^ week of diet and desmin signal increased progressively with diet duration (Supplementary Fig. S4). Zoom-in on a representative hypertrophy area showed that desmin labelling surrounded the area of accumulated retinoid droplets (Fig. 4d). Quantification showed desmin positivity in 80% of the clusters of hypertrophied cells after 9 and 12 weeks of diet.

On SD slices, cRBP1 labelling was observed on cells located in perisinusoidal space (Fig. 4e). On CDAHFD slices, a large, highly localized labelling was present always in areas of cell hypertrophy (Fig. 4f,g). Indeed, 91% of clusters of hypertrophied cells were cRBP1-positive (n = 700 counted areas). Contrary to what was observed with desmin, no cRBP1 labelling was observed on cells lacking retinoid signal. cRBP1 labelling increased progressively with diet time and every cRBP1 positive cell exhibited accumulation of retinoid droplets (Supplementary Fig. S4). Zoom-in of a hypertrophy area showed an intense labelling of cRBP1 in hypertrophied cells closely surrounding retinoid droplets (Fig. 4h).

Under SD, we observed perisinusoidal cells showing both desmin and cRBP1 labelling, characteristic of HSCs. Under CDAHFD, two types of cells were characterized: perisinusoidal desmin positive cells without RF signal and hypertrophied cells in specific areas showing retinoid droplet accumulation and both desmin and cRBP1 labelling. Hence, it can therefore be assumed that retinoid droplets-containing hypertrophied cells in these specific area are of stellate type.

We next investigated the distribution of markers of activated HSCs on CDAHFD and SD-fed mouse liver slices.

### Expression of activated HSCs markers

**Figure 5:**
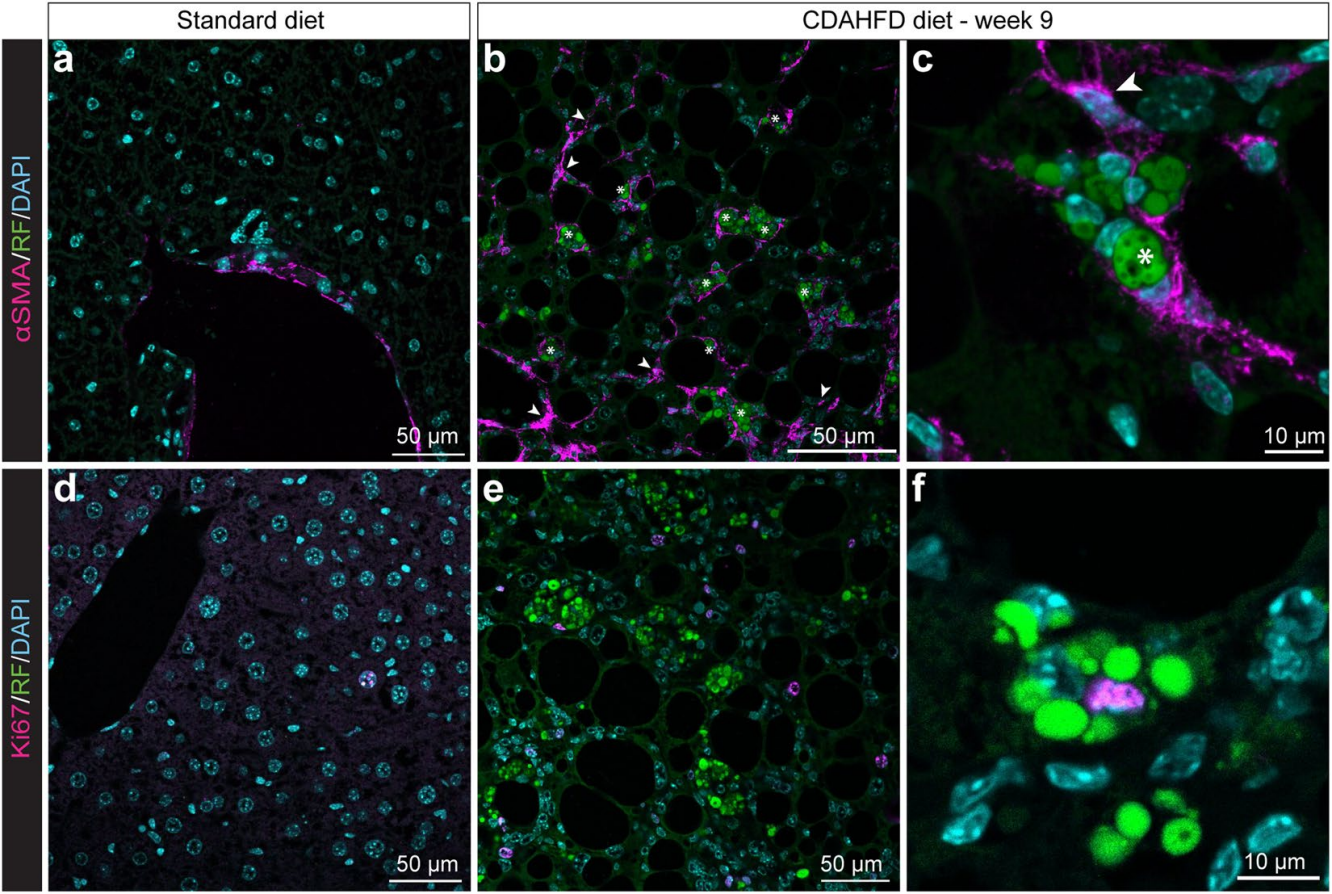
Expression of markers of activated HSCs in hypertrophy areas. (**a–c**) α-SMA labelling of 9 weeks SD- (**a**) and CDAHFD-fed mice (**b,c**) liver slices. Overlap of DAPI, RF and α-SMA signals. Arrowheads: α-SMA-positive cells; stars: α-SMA- and retinoid-positive cells. (**d,e**) Ki67 labelling of 9 weeks SD- (**d**) and CDAHFD-fed mice (**e,f**). Overlap of DAPI, RF and Ki67 signals.

Alpha-smooth muscle actin (α–SMA) is a widely accepted marker of activated HSC^15^. On SD-fed mouse liver slices, α-SMA labelling was only detected around the veins (Fig. 5a), whereas for CDAHFD-fed mice, a significant labelling was detected throughout parenchyma (Fig. 5b). α-SMA labelling in the parenchyma was observed from the 3^rd^ week of diet and increased progressively under diet (Supplementary Fig. S4). Zoom-in on an area of hypertrophy highlighted that α-SMA labelling surrounds exactly the shape of the droplets, indicating the simultaneous presence of α-SMA and retinol droplets in the hypertrophied cells (Figs. 5c and S4D). We quantified 92.5% of clusters of hypertrophied cells surrounded with α-SMA labelling at 12 weeks of diet (n > 200 events). Moreover, as we observed for desmin, some α-SMA positive cells were retinoid signal-free. We then investigated the proliferative state of hypertrophied cells using Ki67 labelling. For SD-fed mice, some rare positive nuclei were observed (Fig. 5d). For CDAHFD-fed mice, Ki67 labelling was much more frequent and seen both in the parenchyma and in areas of cell hypertrophy, demonstrating the regenerative state of the liver upon injury (Fig. 5e). Ki67-positive cells were however less common in areas of hypertrophy, accounting for 14% of labelled cells at 9 and 12 weeks of diet. Magnified imaging of hypertrophied cells showed a cell ongoing division surrounded by retinoid droplets (Fig. 5f).

To conclude on immunofluorescence experiments, cells localized on hypertrophy areas presented both the characteristics of non-activated HSCs as they kept their retinoid storage capacity and were desmin and cRBP1 positive, and the characteristics of activated HSCs as they expressed α-SMA and were proliferating. Moreover, we found that, in the liver of CDAHFD-fed mice, two populations of HSCs coexist, one population of “isolated” activated stellate cells expressing desmin and α-SMA and one population, accumulated in hypertrophy areas that were neither activated nor quiescent and presents an intermediary phenotype with exacerbated number of retinoid storage droplets. To our knowledge, this phenomenon has never been described in a liver fibrosis context, neither in human fibrosis nor in animal models of fibrosis.

### HSC hypertrophy is not model-dependent

**Figure 6:**
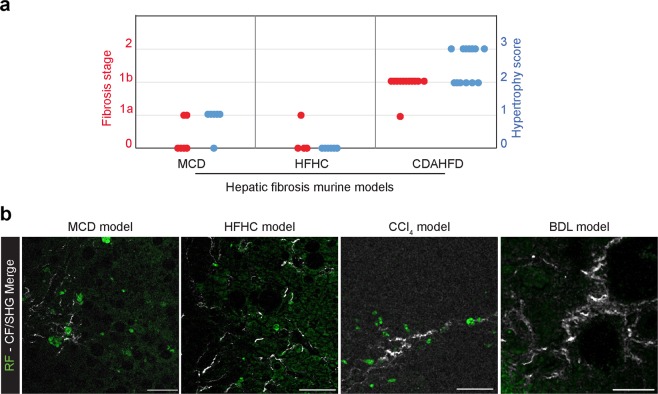
Hypertrophy of HSC is not model-dependent. (**a**) Liver fibrosis (red dots) and HSC hypertrophy scores (blue dots) on 9 weeks MCD diet-fed mice, 16 weeks HFHC diet-fed mice and 22 weeks CDAHFD-fed mice. (**b**) Merge of retinoid fluorescence (RF) imaged by fluorescence and collagen fibers imaged by second harmonic generation (CF/SHG) microscopy on liver section of 9 weeks MCD-, 16 weeks HFHC-, BDL- and CCl_4_- treated mice. Scale bar: 50 µm.

We next investigated the presence of hypertrophied HSCs on two other murine metabolic models of hepatic fibrosis by submitting mice to Methionine and Choline Deficient diet (MCD) for 9 weeks or High-Fat and High-Carbohydrate (HFHC) diet for 16 weeks which are the typical maximal times of diet to obtain fibrosis using these models^10,16^ (Figs. 6a and S5). After 9 weeks of MCD diet, few mice (2/6) showed a light fibrosis (score 1a) while a majority of them showed a moderate form of HSC hypertrophy (5/6). After 16 weeks of HFHC diet, even less mice (1/6) showed a light fibrosis (score 1a) and no HSC hypertrophy was observed (Fig. 6a).

Multi-photon analysis showed that retinoid hypertrophy (RF signal) was also detectable on liver slices of MCD and HFHC diets-fed mice and these hypertrophy areas were always associated with collagen deposits, detectable thanks to SHG (Fig. 6b). Moreover, we also performed multi-photon microscopy analysis of liver sections from mice treated with CCl_4_ or having undergone bile duct ligation (BDL). Interestingly, we found no retinoid fluorescence accumulation on BDL- mice liver slices, neither in the parenchyma, nor in collagen deposit areas. On the other hand, on liver slices of CCl_4_-treated mice, we found the presence of retinoid fluorescence accumulation at the level of collagen deposits (Fig. 6b).

Taken together, these results show that the phenomenon of HSC hypertrophy is not model-dependent.

### HSC hypertrophy is observed in human liver fibrosis

**Figure 7:**
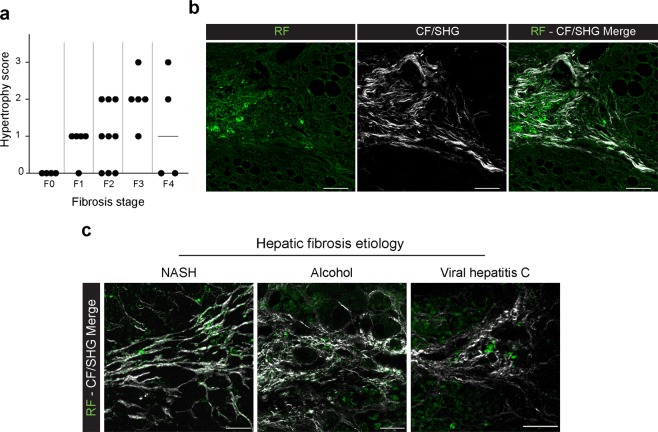
Hypertrophy of HSC is observed in human hepatic fibrosis. (**a**) Liver fibrosis and HSC hypertrophy scores on 27 human liver biopsies from FIBROTA cohort. (**b**) Representative images of collocation of retinoid droplets imaged by fluorescence (RF) and collagen by second harmonic generation (CF/SHG) signal on human liver biopsy from FIBROTA cohort exhibiting F3 liver fibrosis. (**c**) Merge of retinoid fluorescence (RF) and collagen fibers (CF/SHG) imaged by fluorescence and second harmonic generation microscopy on human liver biopsy with fibrosis due to different etiologies: NASH (F2), Viral Hepatitis C (F2) and alcoholism (F4). Scale bar: 50 µm.

To go further, we analyzed human liver biopsies to detect the potential presence of hypertrophied HSC. Human liver biopsies obtained from severely obese patients and presenting different levels of hepatic fibrosis (27 human biopsies ranging from F0 to F4, n ≥ 4 per stage, Supplementary Table 1) were analyzed by confocal microscopy to specifically detect the RF signal of HSC hypertrophied cells through fluorescent spectral analysis. A score of human HSC hypertrophy was set to quantify this occurrence by observation of the retinoid fluorescence signal of the whole biopsy at 200x magnification, as follows: 0 = no visible hypertrophy, 1 = few hypertrophied cells are visible, 2 = 2 to 5 hypertrophied cells areas are observed, 3 = more than 5 hypertrophied cells area are visible. The Spearman correlation coefficient was then used to compare the fibrosis stage established by pathologists and hypertrophy scores (n = 27) and showed that hypertrophy score had a significant positive relationship with fibrosis stage (r = 0.48, p = 0.01), as can be seen in Fig. 7a. Samples were also analyzed by multi-photon microscopy on a blinded manner for RF and CF/SHG signals, showing that the high RF signal was closely located to collagen on every slice presenting a score of fibrosis ≥ F1 (Fig. 7b).

Moreover, we extend our study to other human liver fibrosis etiologies and carried out a fluorescence microscopy analysis on liver sections from patients presenting a hepatic fibrosis due to NASH (n = 4, with F0, F2 or F3 fibrosis), chronic hepatitis C (VHC, n = 7, with F2 or F3 fibrosis) or excessive alcohol consumption (n = 5 with F4 fibrosis). In all cases presenting a fibrosis score greater than or equal to F1, we observed the presence of hypertrophied fluorescent cells near deposits of collagen fibers (Fig. 7c).

Altogether these results show that the phenomenon of HSC hypertrophy is neither model- nor species-dependent. It is found in all murine models tested associated with metabolic fibrosis and also in human tissue with collagen deposit. Frequency of HSC hypertrophy is related to the degree of fibrosis.

## Discussion

In this work, we showed the existence of clusters of hypertrophied HSCs, overloaded with retinoids in their cytoplasmic droplets, in the liver of mice with metabolic liver fibrosis and highlighted the presence of these cells in liver biopsies of patients exhibiting hepatic fibrosis.

We first demonstrated that hypertrophied cells were indeed overloaded with retinoids through a fluorescence spectral study showing that the hypertrophied cells’ droplet spectrum was identical to that obtained on droplets of primary murine HSCs from normal liver. This phenomenon of cytoplasmic retinoid accumulation is usually associated with hypervitaminosis A^17^. Here, the diets (SD, CDAHFD, MCD or HFHC) given to mice contained a standard amount of vitamin A found in rodent diets. Moreover, the measure of total retinoids (retinyl ester and retinol) showed that the overall amount of retinoids in the liver of CDAHFD-fed mice was always lower than in SD-fed mice. This result is consistent with previous studies showing that in experimental models of liver fibrosis and in NASH patient livers, the total amount of liver retinoids tends to decrease^18,19^. Considering the total liver assay result, it can be proposed that although there are less retinoids globally in the liver of CDAHFD-fed mice, a spatial redistribution of retinoids occurs leading to the hypertrophy of some HSCs that have kept their capacity to store retinoids.

We have observed in the literature the occurrence of similar hypertrophied cells (although not expressly mentioned as such) in various models of fibrosis ranging from the toxic CCl_4_ model of liver fibrosis, (Figure 9 in^20^) to nutritional models of fibrosis such as MCD model (Fig. 3 in^21^), or its derivatives like CDAHFD (Figure 8 in^11^), CDAA (Choline-deficient amino acid, Fig. 2 in^22^), and also in a mixed toxic and nutritional model, i.e. STAM mice (streptozotocin injection combined with high-fat diet^23^). Although clearly visible in the figures showing sections of injured liver, the presence of these areas of hypertrophy has not been described by the various authors. For our part, we also showed the presence of these clusters of hypertrophied cells in three other liver fibrosis models in mice (MCD, HFHC and CCl_4_). Interestingly, no retinoid fluorescence accumulation was found on BDL-mice liver slices. BDL is a cholestatic model of liver injury for which fibrosis is mostly originating from periportal myofibroblasts contrary to diet-induced and CCl_4_ models for which fibrosis originates mostly from hepatic stellate cells^4,24^. Thereby, the absence of hypertrophied HSC in BDL-related fibrosis liver slice is probably due to the fact that HSCs are not as involved in the BDL-induced fibrogenesis than in the diet-induced or CCl_4_-induced fibrosis.

Therefore, as we found hypertrophied HSCs in mice liver sections, but also in human liver sections with fibrosis it can be concluded that the presence of hypertrophied cells neither depends on the model nor on the species.

We also showed in CDAHFD-fed mice that HSC hypertrophy was closely related to fibrosis, in terms of onset and growth kinetics, as well as in terms of localization within the liver tissue. This result has been made possible by the combination of second harmonic generation and fluorescence microscopy analysis. These techniques are much more sensitive than conventional histological staining methods, requiring no treatment of the liver slice but more sophisticated equipment. Using these microscopy techniques on two mouse models of diet-induced liver fibrosis causing different levels of fibrosis (moderate for MCD and very modest for HFHC), we showed that HSC hypertrophy was associated with fibrosis. Similarly, biopsies of obese patients with liver fibrosis showed a significant correlation between the grade of fibrosis and the presence of HSC hypertrophy as well as collocation of both phenomena. Moreover, hypertrophied HSCs were observed in CCl_4_ mice liver sections and in human liver sections from etiologies without steatohepatitis (NASH, VHC or alcoholism). These results strengthen our hypothesis that HSC hypertrophy is linked with HSC-induced fibrosis rather than with steatohepatitis.

The occurrence of HSCs containing large amounts of retinoids in the vicinity of the collagen deposition areas seems in contradiction with the well-established fact that, when HSCs become activated and produce collagen, they lose their retinoid droplets. However, it has not been yet unequivocally determined whether the loss of HSC lipid droplets is a cause or a consequence of their activation. For example, a recent study showed that HSCs activated *in vitro* or *in vivo* after liver injury (by bile duct ligation) retained their droplets with a decrease in their retinoid content occurring only after the onset of cell transdifferentiation^25^. Another study suggested that the absence of retinoid-containing droplets in HSCs (in LRAT-deficient mice) did not cause spontaneous fibrosis, nor did it promote fibrosis induced by bile duct ligation or CCl_4_ administration^26^. Thus, although the loss of HSCs droplets is a widely recognized hallmark of liver fibrosis development, it remains to be established at the molecular level why and how these droplets are lost.

The characterization of the cells forming the hypertrophy clusters, using HSC specific markers immunolabelling, suggested that several HSC populations coexist in these areas. Indeed, we observed that these clusters of cells exhibited the expression of (i) a HSC-specific marker, regardless of their status, such as desmin, (ii) a marker linked to the storage process of retinoids, such as cRBP1, (iii) a specific marker of activated HSC such as α-SMA, and (iv) a proliferation marker, such as Ki67. Collocation of these different markers within hypertrophy clusters can be explained by the coexistence of several HSC populations in these particular areas. HSCs are versatile cells in their ability to acquire different phenotypes during liver injury: quiescent, activated, dedifferentiated in myofibroblasts. Even in a healthy and uninjured mouse liver, the HSC population has been reported to be heterogeneous, with a subset of the total HSC population expressing markers of early HSC activation^19^. This leads us to propose that during the onset of liver fibrosis, a reorganization of the tissue occurs, accompanied by heterogeneous storage of retinoids, giving rise to a particular phenotype of hypertrophied HSCs.

The next challenge will be to decipher the underlying mechanisms and signals that lead to HSC hypertrophy. We hypothesize that the onset of hypertrophied HSC is probably due to fairly complex signals that could together involve several types of liver cells and the state of the surrounding extracellular matrix. This is why it seems to us that to identify these mechanisms and signals, studies based on organoid cultures reproducing the injured liver microenvironment will be necessary. Another challenge will be to assess the consequences of hypertrophy on the known functions of HSC. This will require isolating hypertrophied HSCs, adapting the protocols that have been designed for non-hypertrophied HSCs and non-steatotic livers. As the principle of separation of HSCs is based on their lower density compared with other hepatic cells, the presence of hepatocytes loaded with lipids in steatotic liver can interfere with the purity of the preparation. The hypertrophied HSC isolation protocol from CDAHFD-fed mice is being developed in our lab.

In conclusion, the existence of a particular hypertrophied phenotype of HSCs closely related to liver fibrosis is of great interest for the study of the role of HSCs in the process of fibrogenesis. This interest also comes from the fact that this phenotype appears in an *in vivo* context with both inflammation and steatosis. Further characterization of these hypertrophied HSCs would shed light on the mechanisms of HSC activation but also would allow the identification of genes potentially involved in fibrogenesis which would constitute targets for a therapeutic strategy based on RNA interference. Thus, by administration of specific siRNAs, the silencing of these genes could induce an anti-fibrotic effect.

## Material and Methods

### Animals and experimental design

6 week-old male C57BL/6 mice (Janvier Labs Inc., Rennes, France) were housed in cages (5 per cage) and were kept at 23 +/− 3 °C with a 12:12 h light/dark cycle. 90 mice were divided into 2 experimental groups and fed either a standard diet (SD, Specific Diet Services, England 841201, vitamin A content 8000 UI/kg) or a choline‐deficient, L‐amino‐acid‐defined, high‐fat diet with 0.1% methionine (CDAHFD, Safe Diets, France U8958P Version 0247, vitamin A content 5280 UI/kg) for 22 weeks. Two other groups of 15 mice each were fed either with a methionine and choline deficient diet (MCD, Safe Diets, MCD AIN 76, vitamin A content 4424 UI/kg) for 9 weeks, or with a High Fat and High Carbohydrate diet (HFHC, Safe Diets, 260HF, vitamin A content 5207 UI/kg) plus water supplemented by fructose (23.1 g/L) and sucrose (18.9 g/L) for 16 weeks. All animal experiments were approved by the Ethics Committee for Experimentation of Paris Descartes University and authorized by the French Ministry of National Education, Higher Education and Research (APAFiS #4029-2016020513385222v1).

During the experimental period, mice body weight was recorded once a week. At each time point mice were deeply anesthetized (ketamine/xylazine 100/10 mg/kg, *i.p*.) during the light cycle, for blood collection by cardiac puncture and then euthanized by cervical dislocation. The liver was rapidly excised and weighed, the different lobes were separated, fixed in 4% buffered formaldehyde solution for histology analysis or snap frozen for biochemical analyses.

Paraffin embedded liver sections from mice treated with CCl_4_ or having undergone bile duct ligation (BDL) were obtained from Dr Foufelle. For CCl_4_-induced liver fibrosis, 8 week old C57Bl/6 mice received intraperitoneal injections of a CCl_4_ solution (1 g CCl_4_ per kg of body weight in olive oil) twice a week for 8 weeks^9^. For BDL, 8 week old C57Bl/6 mice were ligated during 10 days as described previously^27^.

### Human samples

Human liver samples (surgical biopsy samples) were obtained from 27 severely obese patients during their bariatric surgery and presenting several fibrosis stages: F0 (n = 4), F1 (n = 5), F2 (n = 9), F3 (n = 5) and F4 (n = 4). Patients are part of a clinical research protocol (i.e. FIBROTA, number P100503 –IDRCB 2011-A00759-32), which was recorded on the clinical trial website (NCT01655017). 16 human liver samples were also obtained from patients who underwent liver biopsy between 2008 and 2019 for suspicion of NASH (n = 4) or Viral Hepatitis C (n = 7) and alcoholism (n = 5) and referred to the Hepatogastroenterology Department of the Pitié-Salpêtrière Hospital, Paris, France. The biopsies were embedded in paraffin, cut into 3 µm-thick paraffin sections and routinely picrosirius-Hemalun-stained for fibrosis staging or unstained for fluorescence microscopy analysis. For each biopsy, the stage of fibrosis (F from F0 to F4) was assessed according to NASH-CRN scoring system^28^ with the single modification of pooling the three substages (1a, 1b and 1c) into a single F1 score, or according to METAVIR score in patients with other causes of liver fibrosis.

The study was conducted in accordance with the Helsinki Declaration. Human data and biosample collection were approved by the Ethics Committee (CPP Ile-de-France 1). All subjects provided written informed consent before liver biopsy or when included in the Fibrota clinical trial (NCT01655017).

### Plasma biomarker assays

Plasma biomarker assays were performed with an Olympus AU 400 multiparameter, in the Biochemistry Platform of Centre de Recherche sur l’Inflammation (CRI) CNRS - UMR 1149 Université Paris Diderot.

### Histology

Paraffin embedded liver sections (4 µm thick) were stained with hematoxylin and eosin (H&E) or Sirius red and scored in a blinded manner for the degree of steatosis, inflammation and fibrosis by an experienced pathologist. Steatosis was graded as follows based on the average area (%) of fat droplets: 0, 0%; 1, 1 to 33%; 2, 34 to 66%; 3, 67 to 100%. Inflammation was graded based on the number of inflammatory foci as follows: 0: none; 1: <2 foci per lobule; 2: between 2 and 4 foci per lobule; 3: >4 foci per lobule. The staging of hepatic fibrosis was classified into stages 0 to 4 (stage 0: none; stage 1a: mild centrilobular and perisinusoidal fibrosis; stage 1b: moderate centrilobular fibrosis; 1c: portal fibrosis; stage 2: severe, perisinusoidal and periportal fibrosis; stage 3: bridging fibrosis; and stage 4: cirrhosis).

### Immunohistochemistry

Antibodies (Abcam) against desmin (ab15200; 1/200), c-RBP1 (ab154881; 1/500), α-SMA (ab124964, 1/1000) and Ki67 (ab16667; 1/500) were used for immunohistochemistry on 4 µm paraffin liver sections previously dewaxed and unmasked in sodium citrate buffer (0.1 M; pH 6). AlexaFluor647 conjugated secondary antibody (Fisher Scientific, A21244; 1/500) was then incubated for 1 h at RT and nuclei were stained using 4′,6-diamidino-2-phenylindole (DAPI; 0.3 µg/mL, Sigma-Aldrich, D1388). The slides were then examined under confocal fluorescence microscopy, (SP8 Leica Microsystems) at 400X magnification. Images were acquired using the software Leica Acquisition System X and treated using open source FiJi software.

### Multiphotonic and second harmonic generation microscopy

Multiphotonic microscopy was performed using an LSM 710NLO laser-scanning microscope (Zeiss) equipped with a 20 × dry objective with numerical aperture of 0.8 (Achroplan; Zeiss). The whole tissue sections were entirely scanned by tiling (with an overlap of 10%) multiple 512 × 512 pixel frames. Each tissue section was also scanned for a Z-stack of 3 µm steps with no image average. The Chameleon Ultra Ti:Sapphire laser (Coherent) was tuned at 810 nm. Signals were collected using non-descanned detectors (Zeiss). The second harmonic generation (SHG) signal was detected through a bandwidth filter 395 to 425 nm of a non-descanned detector in a backscattering geometry. Simultaneously, the retinoid fluorescence (RF) signal was detected through a bandwidth filter 500 to 550 nm of a non-descanned detector in a backscattering geometry. The quantitative analysis of multiphoton microscopy images was made through a home-made automated image analysis workflow implemented in Fiji (NIH, Bethesda, MD, USA) environment as a macro.

### Microscopic spectral analysis

Images have been generated on a SP8 confocal microscope (Leica) in a spectral detection mode. Samples were excited either with a 405 nm laser and the emission signal was detected between 420 nm and 650 nm with a spectral window of 5 nm or with a 480 nm laser and emission signal was detected between 490 nm and 790 nm. By selecting a region of interest on the background or on the retinoid droplets signal, we generated specific spectra for the tissue background and for the retinoids. For each sample (primary HSC, mice liver tissue, human biopsies) we took spectral acquisition images and performed a spectral separation, using the Dye Separation tool of confocal software (Leica Application Software X) based on the specific signal of retinoids generated (HSC primary cells) previously. This allowed us to obtain both the fluorescence emission spectra and the separate images of tissue autofluorescence (background) and retinoid signal on all samples analyzed. For human biopsy image analysis, we carried out a spectral separation of retinoid signal based on the specific spectra of primary HSC retinoids, which allowed us to confirm that the fluorescent signal observed was specific for retinoids.

### Liver retinoid content measurement by high-performance liquid chromatography (HPLC)

40–50 mg of frozen liver tissue were homogenized in PBS (phosphate buffered saline) using a Precellys homogenizer (CK14 beads, 03961-1-0032, Ozyme, France). In order to monitor the extraction yields, 2.5 µg of internal standard (IS, retinyl acetate (Sigma; R4632-1G) in ethanol) were added to each sample. Samples were homogenized by sonication for 2 min at RT using an ultrasonic sonifier (Duty cycle: 10%; Output control: 1. Branson 450 Sonifier - Analog Cell Disruptor). Retinoids present in the homogenates were extracted 5 times with cyclohexane. The cyclohexane phases containing retinoids were then evaporated using a speedVac™ for 30 minutes. Retinoid analysis was performed by reverse-phase chromatography on a HPLC system (Waters Symmetry® 150 × 4.6 mm, C18, 5 μm column) at a flow rate of 1.5 ml/ min, using 70% acetonitrile, 15% methanol, 15% methylene chloride as the running solvent for 10 min, followed by 89% acetonitrile, 11% water, 0.1% formic acid for 20 min. Injection volume was 10 µL for all samples. Retinoids were identified and quantified by UV absorbance at 325 nm. Stock standard ethanolic solutions of retinol (Alfa Aesar; J62079), retinyl acetate and retinyl palmitate (Alfa Aesar; J63022) were prepared and used for calibration curves determination (see an example of HPLC chromatogram in Supplementary Fig. S3). Quantification was performed as follows: for each sample, extraction yield was assessed using the retinyl acetate calibration curve. Retinol and retinyl esters were quantified according to retinol and retinyl palmitate calibration curves, respectively, and then corrected according to the determined extraction yield to obtain the actual quantity in each sample.

### Primary murine HSC purification

Murine HSCs were isolated from standard diet-fed mice as follows. Briefly, mice were deeply anesthetized with a lethal dose of anaesthetics (ketamine/xylazine 200/20 mg/kg, i.p). Then a laparotomy and an *in situ* perfusion through the superior vena cava were performed with pre-heated (37 °C) perfusion buffer (25 mM HEPES, 1.2 mM KH_2_PO_4_, 2.3 mM KCl, 86 mM NaCl, 0.5 mM EGTA, pH 7.6). Then the portal vein was cut and perfusion continued with 45 ml of perfusion buffer at a flow rate of 4 ml/min. Liver perfusion was followed with 30 mL of digestion buffer (25 mM HEPES, 2.3 mM KCl, 86 mM NaCl, 5 mM CaCl_2_, pH 7.6) containing Collagenase type I (Gibco 17100-017, 205 U/mg, 1 mg/ml) and Dispase (Gibco 17105-041, 1.79U/mg 0.56 mg/ml). The liver was then removed, cut in small pieces and incubated in 20 ml of digestion buffer at 37 °C for 20 minutes. The digested liver suspension was filtered through a 70 µm cell strainer and washed with Gey’s Balanced Salt Solution (GBSS, 0.4 mM Na_2_HPO_4_, 2.7 mM NaHCO_3_, 0.2 mM KH_2_PO_4_, 137 mM NaCl, 5 mM KCl, 1 mM MgCl2, 0.3 mM MgSO_4_, 5.5 mM Glucose, 2 mM CaCl_2_, pH 7.4). HSCs were isolated from the resulting cell suspension by a Nycodenz gradient centrifugation. HSCs were collected at the interface between GBSS buffer and cell-Nycodenz solution, washed and seeded on uncoated plastic tissue culture dishes in DMEM supplemented with 10% FBS (Fetal Bovine Serum), and antibiotics (1% Penicillin/streptomycin), and then cultured in 5% CO_2_ humidified atmosphere at 37 °C. Growth medium was changed daily.

### Statistical analysis

Prism 6.0 (GraphPad Software Inc.©, USA) was used for statistical analysis. Data were analyzed by Mann–Whitney test or Kruskal-Wallis test after being assessed for non-normal distribution. Spearman’s rank correlation coefficient was calculated in Excel. The number of repetitions and statistical tests used for each data set is indicated in the figure legends. Statistical significance is indicated on the graphs with asterisks (*p < 0.05; **p < 0.01; ***p < 0.005; ****p < 0.0005).

## Supplementary information

Supplementary information.
