# Supplementary material for: Hepatic stellate cell hypertrophy is associated with metabolic liver fibrosis
Source: Sci Rep. 2020 Mar 2;10:3850. doi: 10.1038/s41598-020-60615-0 (PMC7052210; doi:10.1038/s41598-020-60615-0)
Supplement: Supplementary file 1 — Supplementary information. [file 41598_2020_60615_MOESM1_ESM.pdf]

Supplementary data

Hepatic stellate cell hypertrophy is associated with metabolic liver fibrosis

Céline Hoffmann, Nour El Houda Djerir, Anne Danckaert, Julien Fernandes, Pascal Roux, Christine Charrueau, Anne-Marie Lachagès, Frédéric Charlotte, Isabelle Brocheriou, Karine Clément, Judith Aron-Wisnewsky, Fabienne Foufelle, Vlad Ratziu, Bernard Hainque, Dominique Bonnefont-Rousselot, Pascal Bigey, Virginie Escriou

Supplementary Figure S1

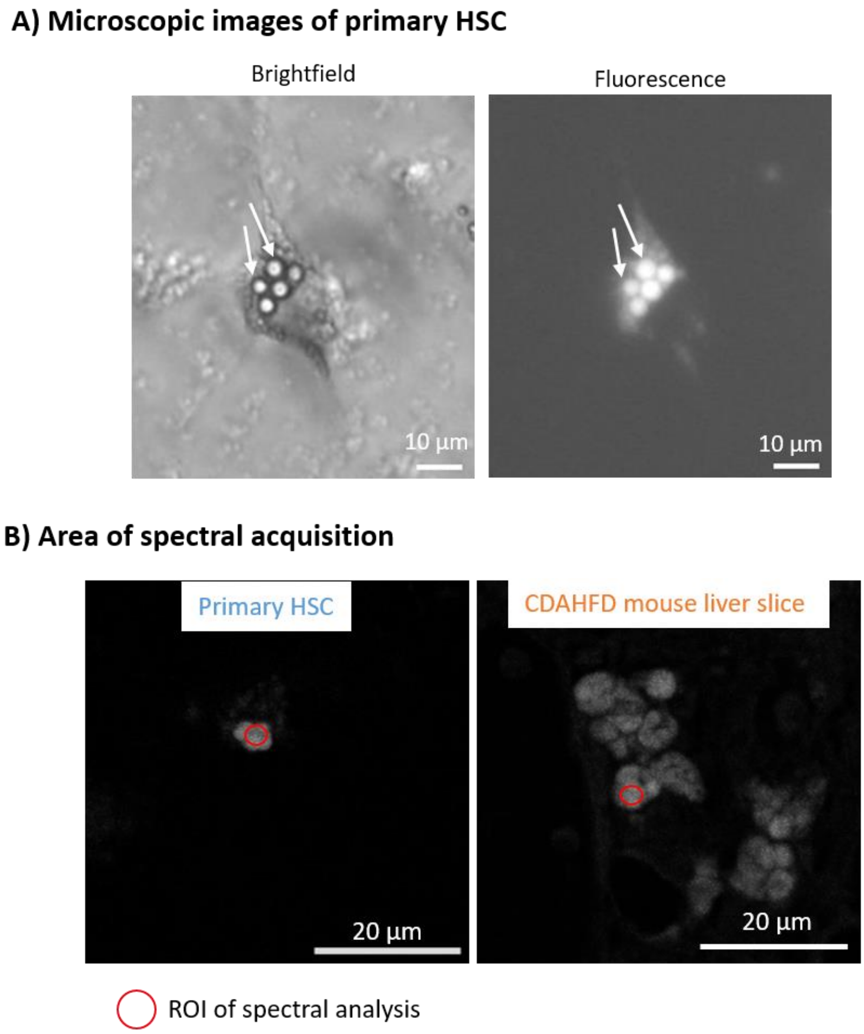

Supplementary Figure S1. Spectral analysis of retinoids in murine primary hepatic stellate cells and mouse liver tissue slice. A) Microscopic images of murine primary HSC purified from SD-fed mouse under brightfield illumination and fluorescent excitation (λ_{exc}= 405 nm) exhibiting droplets of retinoids (arrows). B) ROI where the emission spectra of main figure 2F were extracted is represented as a red circle.

Supplementary Figure S2

A)

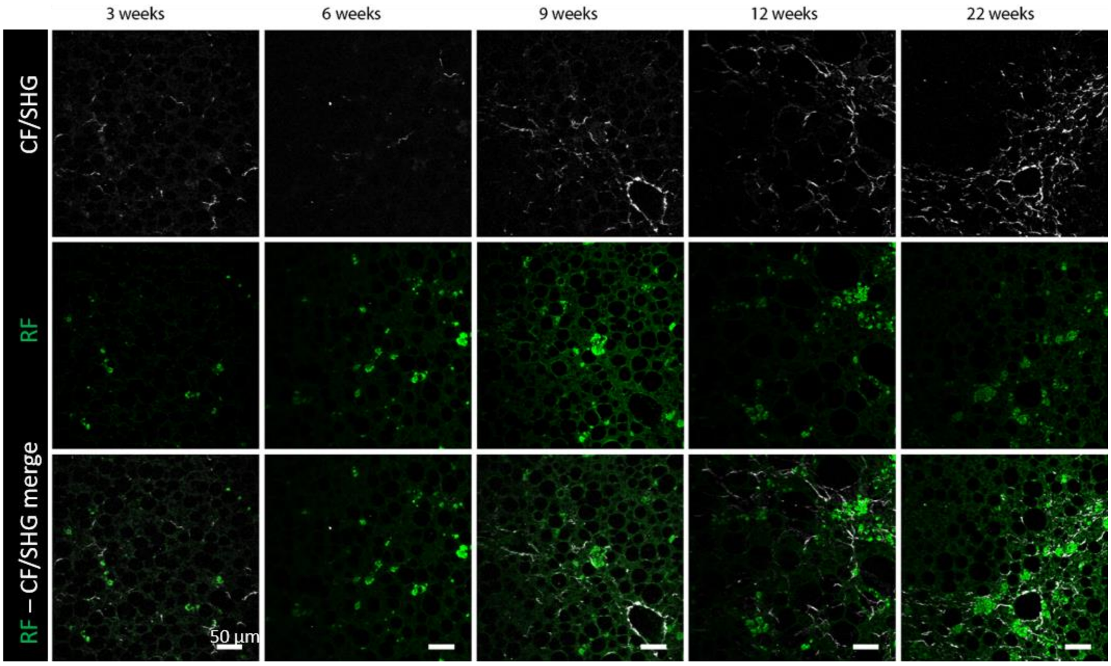

B)

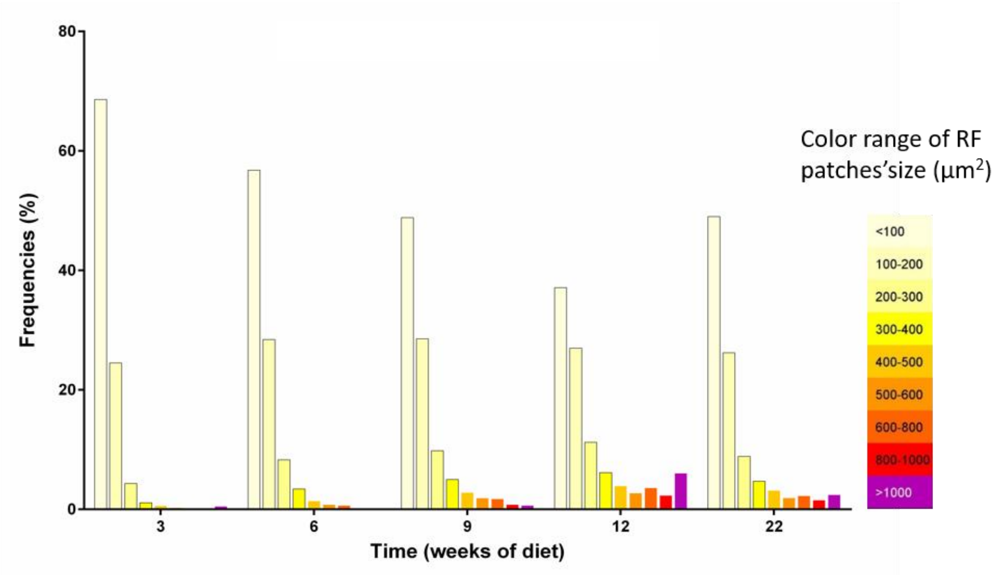

Supplementary Figure S2. Kinetics of collagen and retinoid accumulation on liver slices of CDAHFD-fed mice as a function of time. A) Kinetics of collagen and retinoids analyzed by fluorescence microscopy and second harmonic generation microscopy. Representative images of collagen accumulation recorded by second harmonic generation microscopy (CF/SHG signal - top panels) and retinoid fluorescence (RF) signals imaged by fluorescence (middle panels) of mice liver slices after 3, 6, 9, 12 and 22 weeks of CDAHFD. Bottom panel is a merge of the two previous signals. Scale bars: 50µm. B) Kinetics of retinoid accumulation. Frequency histogram of RF patches'size as a function of CDAHFD time. Color range of RF patches 'size is expressed in µm².

Supplementary Figure S3

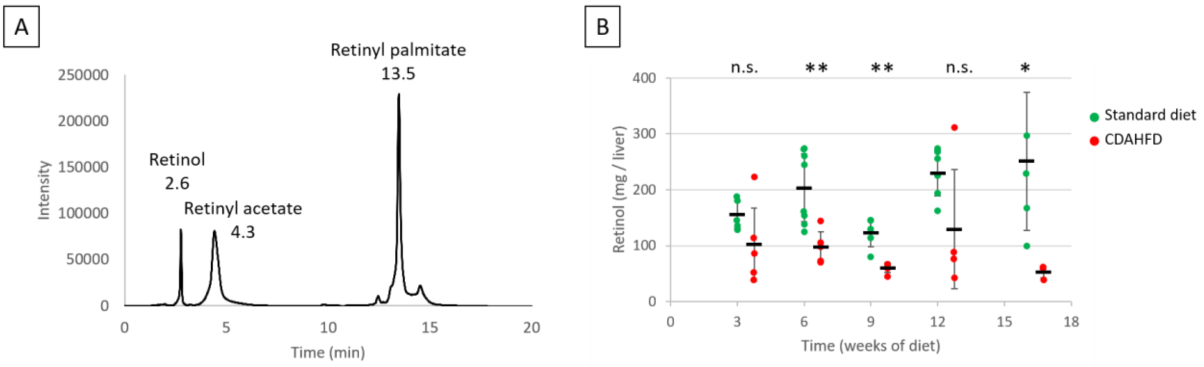

Supplementary Figure S3. Retinol quantification in liver from SD- and CDAHFD-fed mice by HPLC. A) Example of HPLC spectrum of mixture of retinoid standards. B) Total liver content in retinol as a function of time in the liver of SD- (green dots) or CDAHFD-fed mice (red dots) (n=4-7 for each condition). Statistic Mann-Whitney compared SD to CDAHFD at a given time, n.s. not significant, * 0.010 < p < 0.05; ** p < 0.010.

Supplementary Figure S4

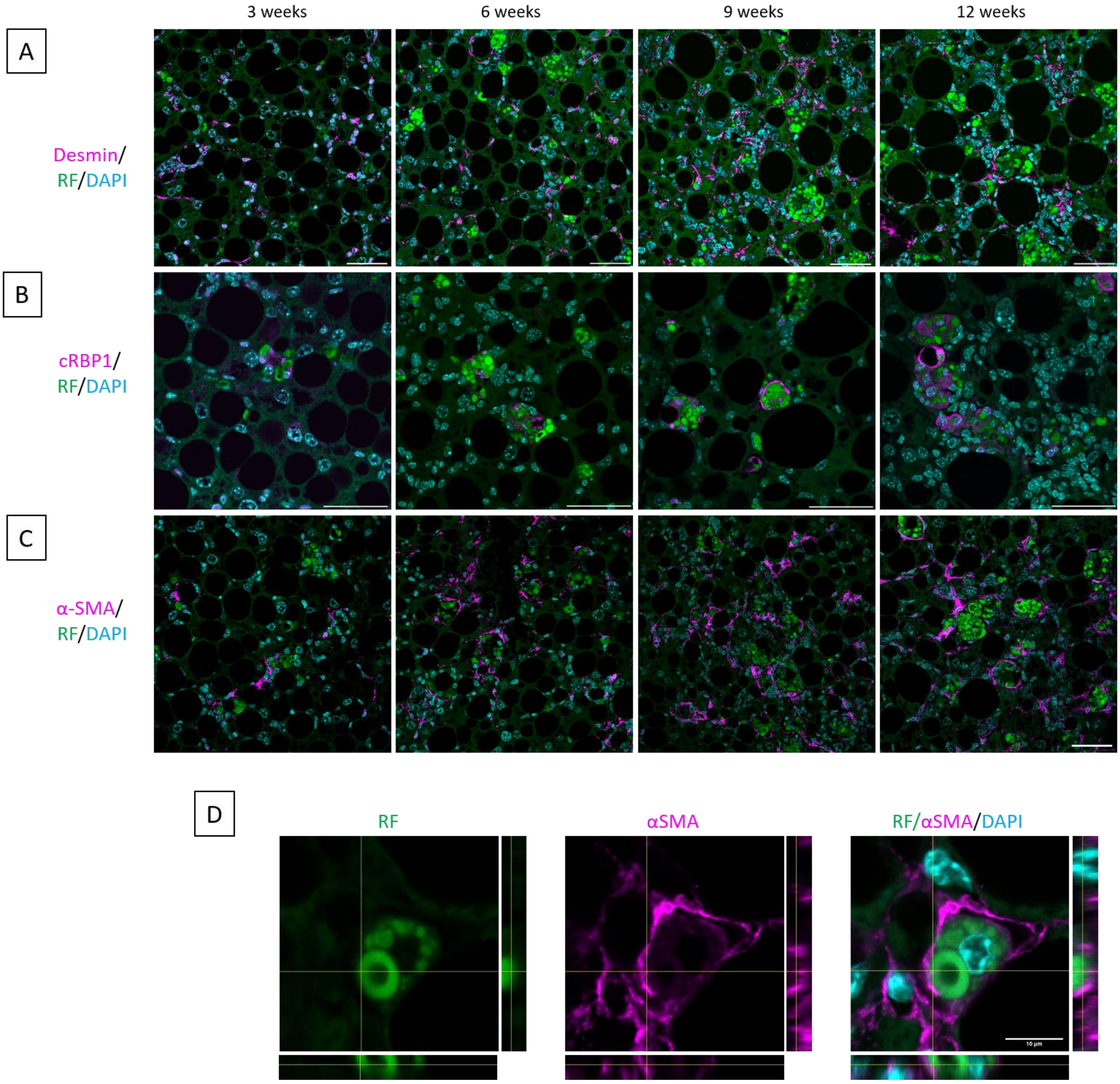

Supplementary Figure S4. Kinetics of hepatic stellate cells markers evolution on liver slices of CDAHFD-fed mice as a function of time. A) Desmin, B) cRBP1 and C) α-SMA labelling of liver slices from CDAHFD-fed mice. Overlap of DAPI, RF (retinoid fluorescence) and A) Desmin, B) cRBP1 and C) α-SMA signals at 3, 6, 9 and 12 weeks of diet. Scale bars = 50 µm. D) Z-stack of α-SMA immunolabelled CDAHFD-fed mice liver slice allowed the imaging hypertrophied HSC in the third spatial dimension and highlighted the colocation of retinoid fluorescence (RF) droplets (left), α-SMA (middle). Overlap of DAPI, RF and α-SMA signals (right). Right panel and bottom panel for each: 4.3 µm-width orthogonal view of the Z stack along the yellow lines. Scale bar: 10 µm

Supplementary Figure S5

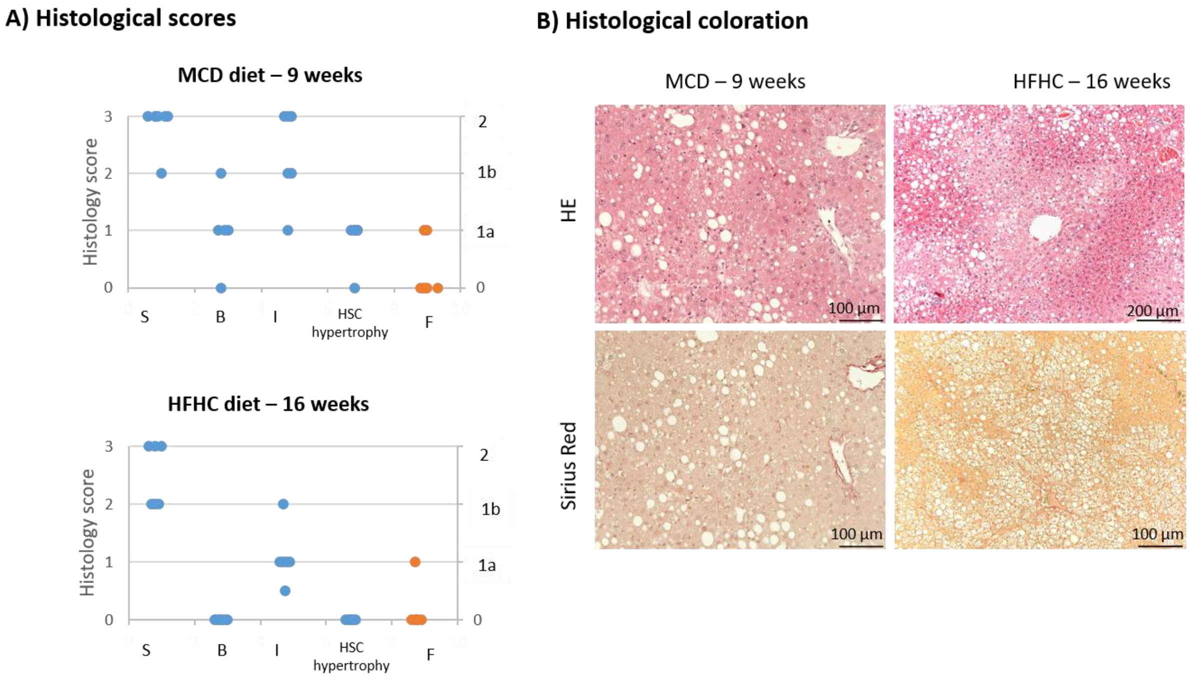

Supplementary Figure S5. Characterization of other liver fibrosis models. A) Histology scores after 9 weeks of MCD diet and 16 weeks of HFHC diet for Steatosis (S), Ballooning (B), Inflammation (I), HSC hypertrophy and Fibrosis (F) (n=6). (B) Histological coloration of liver slices of mice submitted to MCD diet for 9 weeks or HFHC diet for 16 weeks. Hematoxylin/Eosin coloration (top panel) and Sirius Red staining (low panel). Scale bars = 100µm.

Supplementary Table 1

	Median (range)
Number of patients	27
Age at surgery in years	47 (22-67)
Female (%)	80 %
Male (%)	20 %
BMI	43 (35-52)
Markers	
ALT (UI/L)	34 (14-298)
AST (UI/L)	36 (19-117)
Fibrotest score[1]	0.20 (0.03-0.66)
HSC hypertrophy score[2]	1 (0-3)

1. Imbert-Bismut F, Ratziu V, Pieroni L, et al. Biochemical markers of liver fibrosis in patients with hepatitis C virus infection: a prospective study. *Lancet.* 2001;357(9262):1069-1075.

2. Established by us and described in the paragraph "HSC hypertrophy is observed in human liver fibrosis" of the results section.

Supplementary Table 1. Characteristics of patients included in the study. All patients were part of the same clinical research protocol (FIBROTA, number P100503 –IDRCB 2011-A00759-32 recorded on the clinical trial website (NCT01655017)) that included obese patients who have undergone bariatric surgery during which a liver sample was taken.
